# Depression history modulates effects of subthalamic nucleus topography on neuropsychological outcomes of deep brain stimulation for Parkinson’s disease

**DOI:** 10.1038/s41398-022-01978-y

**Published:** 2022-05-27

**Authors:** Ian H. Kratter, Ahmed Jorge, Michael T. Feyder, Ashley C. Whiteman, Yue-fang Chang, Luke C. Henry, Jordan F. Karp, R. Mark Richardson

**Affiliations:** 1 Department of Psychiatry, University of Pittsburgh School of Medicine, Pittsburgh, PA USA; 2 Brain Modulation Laboratory, Department of Neurological Surgery, University of Pittsburgh School of Medicine, Pittsburgh, PA USA; 3 University of Pittsburgh School of Medicine, Pittsburgh, PA USA; 4 Department of Psychiatry, College of Medicine, University of Arizona, Tucson, AZ USA; 5 Department of Neurosurgery, Massachusetts General Hospital, Boston, MA USA; 6 Harvard Medical School, Boston, MA USA; 7 Present Address: Department of Psychiatry and Behavioral Sciences, Stanford University School of Medicine, 401 Quarry Road, Stanford, CA 94305 USA

**Keywords:** Depression, Human behaviour

## Abstract

Patients with psychiatric symptoms, such as depression, anxiety, and visual hallucinations, may be at increased risk for adverse effects following deep brain stimulation of the subthalamic nucleus for Parkinson’s disease, but there have been relatively few studies of associations between locations of chronic stimulation and neuropsychological outcomes. We sought to determine whether psychiatric history modulates associations between stimulation location within the subthalamic nucleus and postoperative affective and cognitive changes. We retrospectively identified 42 patients with Parkinson’s disease who received bilateral subthalamic nucleus deep brain stimulation and who completed both pre- and postoperative neuropsychological testing. Active stimulation contacts were localized in MNI space using Lead-DBS software. Linear discriminant analysis identified vectors maximizing variance in postoperative neuropsychological changes, and Pearson’s correlations were used to assess for linear relationships. Stimulation location was associated with postoperative change for only 3 of the 18 neuropsychological measures. Variation along the superioinferior (z) axis was most influential. Constraining the analysis to patients with a history of depression revealed 10 measures significantly associated with active contact location, primarily related to location along the anterioposterior (y) axis and with worse outcomes associated with more anterior stimulation. Analysis of patients with a history of anxiety revealed 5 measures with location-associated changes without a predominant axis. History of visual hallucinations was not associated with significant findings. Our results suggest that a history of depression may influence the relationship between active contact location and neuropsychological outcomes following subthalamic nucleus deep brain stimulation. These patients may be more sensitive to off-target (nonmotor) stimulation.

## Introduction

Deep brain stimulation (DBS) targeted to the dorsolateral sensorimotor region of the subthalamic nucleus (STN) is a well-established treatment for motor symptoms of Parkinson’s disease (PD) insufficiently controlled by medications [1, 2]. DBS is generally well tolerated, but psychiatric and cognitive adverse effects may occur [3–9], and relatively little is known about the risk factors for such outcomes.

The organization of the STN is notable for partial anatomic and functional segregation, such that subregions most connected to limbic and associative basal ganglia-thalamocortical circuits are located ventromedial and anterior to the sensorimotor region [10, 11]. This topography has motivated investigation into putative relationships between specific stimulation locations and DBS outcomes. Few studies, however, have systematically examined relationships between chronic stimulation location and neuropsychiatric outcomes [12–22], and, among those, fewer still have utilized comprehensive neuropsychological testing (NPT) [13–15, 17].

Depression, anxiety, visual hallucinations (VH), and impulse control behaviors are common psychiatric symptoms of PD [23], and, particularly in the case of depression, may occur prior to disease onset [24, 25]. Such cases featuring early and prominent psychiatric symptoms suggest underlying neurobiological differences. Outside the context of PD, depression is associated with changes in the functional connectivity of multiple large-scale brain networks (for review see [26]), and more recent studies suggest that some of these differences persist even after resolution of the depressive episode [27–30]. Anxiety disorders also are associated with changes in functional connectivity [31], and such differences may be specific for a clinical diagnosis rather than high-worrier traits [32]. Altogether, these differences raise the possibility that individuals with a history of common psychiatric diagnoses may be differentially susceptible to nonmotor effects of off-target DBS. Consideration of psychiatric history, however, has not been included in previous investigations of relationships between stimulation location and neuropsychological outcomes.

Additionally, previous studies of relationships between stimulation location and neuropsychological outcomes have been limited by inadequate ability to reconstruct active contact locations in group space, making comparisons across studies difficult, or differences in crucial variables including stimulation laterality, stimulation duration, medication state during assessment, and means of assessment.

To explore the possibility that psychiatric history modulates relationships between stimulation location and neuropsychological outcomes, and to overcome the aforementioned technical limitations, we used linear discriminant analysis (LDA) machine learning to explore relationships between the active contact location, as mapped into common STN space using a state-of-the-art imaging platform, and changes in neuropsychological measures following STN DBS, in a well-characterized clinical cohort. We then repeated these analyses after dividing the entire cohort into groups based on history of common PD-associated psychiatric symptoms: depression, anxiety, and VH.

## Materials and methods

### Subjects

We retrospectively reviewed electronic medical records of all patients with a diagnosis of PD who underwent STN-DBS implantation by a single surgeon (RMR) between December 2012 and March 2018, in accordance with a protocol approved by the University of Pittsburgh Institutional Review Board. Additional inclusion criteria included bilateral surgery, chronic bilateral active stimulation following activation of the device, and the presence of both pre- and postoperative NPT data. Exclusion criteria were preoperative clinical diagnosis of dementia, Mini Mental Status Exam score<24, or device explant prior to postoperative NPT.

### Chart review

Demographics, relevant clinical data, and stimulation parameters were collected for each subject by an abstractor (IHK) blinded to NPT results. Medications at the time of both pre- and postoperative NPT were reconciled, and levodopa equivalent daily dosages (LEDD) [33] were calculated. The preoperative number of nonmotor medications and Charlson Comorbidity Index [34] were used as measures of medical comorbidity. History of depression was defined as a documented diagnosis of current or past depressive disorder. History of anxiety was defined as a documented diagnosis of current or past generalized anxiety or panic disorder. History of VH was defined as documentation of any history of visual illusion or hallucination outside the setting of delirium. History of impulse control behavior was defined as a documented notable increase from baseline in impulsive or repetitive behaviors. In addition to reviewing all available relevant clinical notes, we also utilized the Epic search function for the terms ‘depressed,’ ‘depression,’ ‘mood,’ ‘anxiety,’ ‘panic,’ ‘hallucination,’ ‘VH,’ ‘illusion,’ ‘shadow,’ ‘impulse,’ ‘compulsive,’ ‘ICD,’ ‘shopping,’ and ‘gambling’ to reduce the likelihood of missing relevant documentation.

### Neuropsychological testing

Prior to analysis we decided to utilize only a subset of the NPT battery, with the goal of assessing all major cognitive domains and common psychiatric symptoms but minimizing the number of statistical comparisons to decrease the risk of type I errors. Based on previously published reports [35–37], we ultimately included 15 cognitive measures: Trails Making Test-B; Color-Word Interference test (Stroop analog from the D-KEFS); WASI-II Matrix Reasoning and Similarities tests; RBANS Semantic Fluency, List Learning, List Recall, Figure Copy, Figure Recall, and Digit Span Forward tests; Boston Naming Test; Wechsler Test of Adult Reading (WTAR); Controlled Oral-Word Association test (F-A-S); and, WAIS Digit Span Backward and Picture Completion tests. Three psychiatric measures were included: the Center for Epidemiologic Studies Depression Scale (CES-D) [38], the Zung Self-Rating Anxiety Scale (SAS) [39], and the Apathy Evaluation Scale (Apathy) [40]. For all cognitive tests, raw scores were converted to psychometric *T* scores (mean=50, standard deviation=10) to control for normative variables (i.e., age, sex, and level of education) and to facilitate direct comparison between pre- and postoperative scores. A final ΔT score was calculated as Δ*T* = *T*_postoperative_–*T*_preoperative_. SAS raw scores were converted to the publisher’s provided “Anxiety Index” prior to subtraction of the preoperative score from the postoperative score. Raw score differences were compared for CES-D and Apathy due to lack of available normalized indices.

### Surgical approach, lead localization, and volume of tissue activation

Presurgical MRI and postoperative MRI or CT were obtained for all patients. Lead implantation was performed as previously described utilizing either microelectrode recording or intraoperative MRI [41]. The deepest contact always was placed at the ventral border of the STN as defined by microelectrode recording or targeted to a depth of −2mm in the z-axis of AC-PC space in intraoperative MRI cases.

DBS electrode localizations and volumes of tissue activated (VTA) were calculated using Lead-DBS software (www.lead-dbs.org) as previously described [42]. Briefly, postoperative images were linearly coregistered to preoperative MRI using Advanced Normalization Tools (ANTs) [43], SPM12 [44], or a hybrid function of the two. The coregistered image sequences were spatially normalized into MNI ICBM 2009b NLIN asymmetric space using the non-linear ANTs SyN Diffeomorphic Mapping approach [43]. A refined affine transform restricted to our subcortical area of interest to account for brain-shift was implemented if visual inspection revealed improved results. Final localization in MNI template space was applied to the DISTAL minimal atlas [45]. With monopolar stimulation, coordinates utilized were that of the active (cathode) contact. With bipolar stimulation, coordinates were taken to be the mean between the cathode and anode contacts. Of note, transformation from individual to group space representation inherently causes some contacts within the STN to appear to reside outside of it due to variation in individual STN shape and size. Additionally, given the STN’s relatively small size, the more superior contacts of the DBS leads tend to reside outside of the STN, and such contacts in the zona incerta have been shown to provide therapeutic benefit [46] and so may have been selected empirically by the programming clinician as the best stimulation location.

VTA was modeled according to the clinically documented stimulation parameters and default Lead-DBS settings, which apply a Finite Element Method-based model to estimate the E-field on a four-compartment tetrahedral mesh and set a gradient vector magnitude threshold at 0.2 V/mm.

### Linear discriminant analysis

We used an LDA machine learning algorithm to identify a vector that maximized variance in outcomes. Specifically, the dependent variable (Δ*T* score) was split into three classes: postoperative worsening (Δ*T* < −5), no postoperative change (−5≤Δ*T*≤5), and postoperative improvement (Δ*T* > 5). The Δ*T* range of 10 for the unchanged class was chosen because it represents one standard deviation, and Δ*T* > 10 (i.e., change greater than one standard deviation) is commonly accepted as the threshold for a clinically significant change. The MNI coordinates (MNI_x_, MNI_y_ and MNI_z_) of the localized active contacts were used as the continuous independent variables. We then derived an LDA model that attempted to reorient the original MNI coordinate axes to a coordinate axis that maximized the variability between the Δ*T* scores in 3D space [47]. The dimension explaining the highest percent of class-to-class variability (LDA_1_, shown in standardized LDA space) was chosen and then transformed back onto the original MNI space as the vector represented in our figures, with the positive direction arbitrarily defined as oriented towards the postoperative improvement class. The proportions of the LDA_1_ vector projected on to each MNI axis also were calculated.

### Statistical analysis

Distributions of continuous variables were checked for normality and then compared with paired or 2-sample t-tests, Welch’s test for data with unequal variances, Wilcoxon rank sum test, or Spearman correlation. Chi-square test was used to compare categorical variables. Association between baseline depression and anxiety symptoms and postoperative change on NPT was evaluated using linear regression to control for baseline symptom severity and then test whether the change score was statistically significantly different from 0. Correlation between postoperative change on each neuropsychological assessment (Δ*T* score) and active contact location along the vector determined by LDA to explain the highest percent of class-to-class variability (LDA_1_, as described further above) was assessed using Pearson’s coefficients. Each brain hemisphere was analyzed separately. Data are presented as mean ± SD unless otherwise noted. To decrease Type I errors, the Benjamini-Hochberg procedure [48] was used with a false discovery rate set at α = 0.10. Analyses were performed in MATLAB R2018B, Qt Console for Python, and SAS 9.4.

## Results

### Cohort description

42 of 157 PD patients treated with STN-DBS met study criteria (26.8%). The most common reason for exclusion was lack of return for recommended postoperative neuropsychological follow-up and NPT (101 of 115, 87.8%; see Fig. S1 for further details). The study cohort predominantly consisted of right-handed, middle-aged, well-educated, Caucasian males who had been diagnosed with PD within the decade prior to surgery but who otherwise were without major medical comorbidity (Table 1). Mean time until NPT follow-up was 1.4 years. Some patients were unable to complete all measures for various reasons (e.g., fatigue, sensoriperceptual problem), but all patients completed at least 15 of the measures both pre- and postoperatively, with the exceptions of 2 preoperatively and 1 postoperatively. One patient’s scores for Digit Span Forward and Digit Span Backward tests were excluded due to poor performance that was judged at the time of assessment to reflect hearing impairment and not cognitive ability. All DBS device models were either Medtronic 3389 (83%) or St. Jude 6172 (17%). Of 84 total implanted leads (42 patients with bilateral DBS), 51 (61%) were delivering monopolar stimulation, whereas 33 (39%) were delivering bipolar stimulation.

**Table 1:** Description of Cohort.

	ALL (*N* = 42)	DEPRESSION (*N* = 12)	ANXIETY (*N* = 15)	VH (*N* = 11)
AGE, YEARS MEAN (SD)	67.6 (7.6)	66.3 (5.8)	64.1 (8.7)*	70.3 (9.5)
SEX NUMBER MALE (%)	30 (71)	10 (83)	10 (67)	7 (64)
HANDEDNESS NUMBER RIGHT-HANDED (%)	41 (98)	12 (100)	15 (100)	11 (100)
EDUCATION, YEARS MEAN (SD)	14.8 (3.0)	16.0 (3.5)	14.8 (2.9)	14.5 (2.7)
DURATION OF DISEASE, YEARS MEAN (SD)	8.9 (5.1)	8.5 (2.6)	9.2 (5.4)	9.1 (4.5)
PRE-OP NON-MOTOR MEDICATIONS MEAN (SD)	4.3 (3.1)	4.8 (2.8)	4.2 (2.6)	4.2 (2.7)
CHARLSON COMORBIDITY INDEX^‡^ MEAN (SD)	2.6 (1.1)	2.3 (0.9)	2.4 (1.4)	2.7 (1.3)
PRE-OP PD MOTOR MEDICATIONS MEAN (SD)	2.6 (1.2)	2.4 (0.9)	2.9 (1.1)	2.8 (1.2)
PRE-OP LEDD, MG/DAY MEAN (SD)	1104 (636)	1297 (785)	1381 (688)*	1238 (547)
PRE-OP UPDRS III^¥^ (OFF) MEAN (SD)	41.1 (12.8)	40.9 (13.6)	42.3 (13.8)	43.8 (14.2)
PRE-OP UPDRS III (OFF-ON), % CHANGE MEAN (SD)	44.8 (16.2)	50.6 (12.7)	40.6 (11.7)	48.0 (12.8)
DURATION BETWEEN NPT, YEARS MEAN (SD)	1.4 (1.2)	1.2 (1.1)	1.4 (1.3)	2.0 (1.6)
POST-OP LEDD, % CHANGE MEAN (SD)	35.8 (41.3)	28.9 (54.9)	46.2 (22.9)	46.2 (28.2)

^*^Indicates a significant difference (*p* < 0.05) between yes/no condition by 2-sample *t*-test. Note that data for ‘no’ conditions for depression, anxiety, and VH are not shown for clarity. ‡ Predicts 10-year survival in patients with multiple comorbidities with increasing values associated with decreased survival. For example, 2 points is associated with predicted 90% likelihood of survival over the next 10 years. ¥ Range 0–56, with increasing values represent worsened PD motor symptoms. Differences do not meet statistical significance unless otherwise noted. VH visual hallucinations. LEDD levodopa equivalent daily dosage. UPDRS Unified Parkinson’s Disease Rating Scale Part III score. NPT neuropsychological testing.

From these 42 patients we identified 12 (29%) with a history of depression, 15 (36%) with a history of anxiety, and 11 (26%) with a history of VH. Descriptive characteristics of these patients were similar, with the exceptions that patients with a history of anxiety were slightly younger and had greater LEDD preoperatively (Table 1). In terms of comorbidity, 5 of the above patients had histories of both depression and anxiety; 2, 3, and 1 of those with a history of VH also had a history of depression, anxiety, or both depression and anxiety, respectively. As theoretical modeling of a sample dataset with LDA indicated that less than 11 total data points was unlikely to produce an accurate model (data not shown), all further analysis was performed without regard to comorbidity. Finally, we identified 6 patients (14%) with a history of impulse control behavior but similarly did not perform further analysis of this subgroup given the low sample size.

We also compared preoperative NPT results among the subgroups. We again found few overall statistical differences, although history of depression was associated with worsened performance in semantic fluency, phonemic fluency, and list learning (Table S1). Notably, patients with history of depression or anxiety were statistically indistinguishable from those without such histories on preoperative measures of depression, anxiety, and apathy.

### Postoperative results

Next, we compared postoperative NPT performance changes in the cohort (Table S2). Statistically significant but clinically mild (<1 SD) declines in two tests of executive function (Trail Making Test-B and Stroop Analogue) and two tests of language (F-A-S and RBANS Semantic Fluency) were noted. We repeated this analysis according to subgroup and found one significant change each for patients with history of depression or history of anxiety, respectively. Patients with a history of VH demonstrated significant declines in four tests.

To determine if preoperative symptoms of depression or anxiety (as opposed to history of these diagnoses) might influence postoperative NPT changes, we repeated the above analyses but used a generalized linear model to control for baseline depression (CES-D score; Table S3) or baseline anxiety (SAS score; Table S4). The same statistically significant differences for the entire cohort, the history of depression subgroup, and the history of anxiety subgroup were identified (compare Table S2 with S3-4), meaning that these changes were not associated with measurements of preoperative burden of depression or anxiety. Controlling for baseline depression or anxiety did identify some distinct postoperative NPT changes in the history of VH subgroup, however.

### Active contact location analysis

**Fig. 1:**
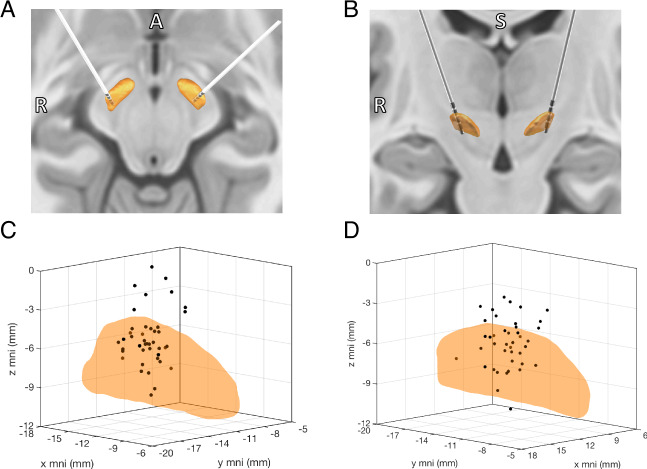
Localization of active DBS contacts. Example localization of STN-DBS electrodes in NMI normalized brain space as viewed in axial (**A**) and coronal (**B**) projections. Locations of the active contact in MNI space for all patients (*N* = 42) at the time of neuropsychological testing for the left (**C**) and right (**D**) STN.

**Fig. 2:**
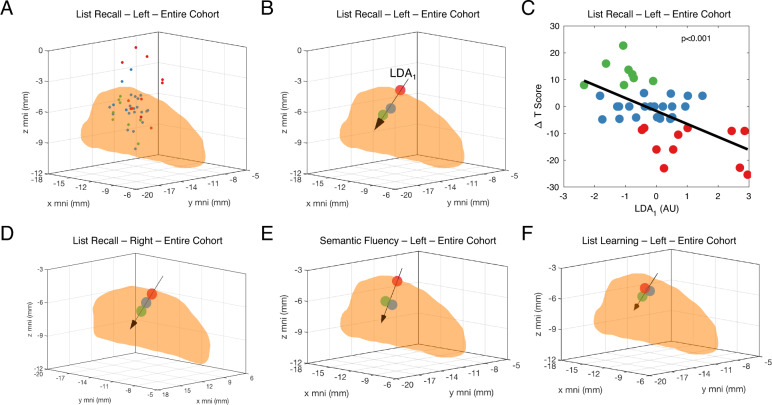
Relationships between active contact location and post-DBS cognitive change for the entire cohort. **A** Active contact location in MNI space for the left STN for all 42 patients at the time of postoperative neuropsychological testing stratified into three classes according to change in postoperative performance (postoperative worsening—red; no postoperative change—blue; postoperative improvement—green) on the list recall task, a measure of memory. **B** Mean contact location in MNI space for each of the three classes is identified with superimposed vector (LDA_1_) that maximizes both the variance of the data and the separation among the classes as determined by linear discriminant analysis (LDA). **C** Plot of change in postoperative test performance in relationship to the axis identified by the LDA vector reveals a significant linear correlation. **D**–**F** Mean contact location in MNI space for each of the three outcome classes is plotted along with the LDA vector for the list recall task for the right STN (**D**), the semantic fluency task, a measure of language, for the left STN (**E**), and the list learning task, another measure of memory, for the left STN (**F**).

We localized the active contact in standardized MNI space for all patients (Fig. 1). Please see the methods section for further details regarding why some contacts appear to reside outside of the STN. Each patient’s contact location in MNI space was then divided into three classes based on postoperative improvement (Δ*T* > + 5, green), worsening (Δ*T* < −5, red), or no change (−5 ≤ Δ*T* ≤5, blue), for each NPT measure (Fig. 2A). Next, we plotted the mean spatial location of each of these classes (larger red, green, and blue circles in Fig. 2B) and used LDA to determine the vector that both best explained ΔT variance and maximized class-to-class variability in MNI coordinate space (LDA_1_ arrow in Fig. 2B). LDA was chosen because it takes class variability into account when reducing dimensionality of a dataset, whereas more traditional principle component analysis does not. Each of the 42 active contact locations (Fig. 2A) then was projected and normalized onto the LDA_1_ axis (Fig. 2B) and plotted in 2D space to assess for linear correlations (Fig. 2C). We repeated this process for each included NPT measure, and the proportion of variance explained by LDA_1_, Pearson’s *r*, uncorrected p-value, and proportion of the LDA_1_ vector projected on to each MNI axis for each measure are listed in Table S5.

As an example, Fig. 2A–C demonstrates the LDA approach to analysis of postoperative change for the list recall task (a verbal memory measure) in relationship to active contact location in the left STN. LDA_1_ explained 92.2% of the total variance for this measure with a Pearson’s *r* of 0.59 (*p* < 0.0001). This means that reducing a contact’s location from 3D space to its location along a single axis provided a powerful descriptor for the association between stimulation location and postoperative change.

We identified additional significant relationships between left STN contact location and postoperative change in list learning and semantic fluency, and right STN contact location and postoperative change in list recall (Fig. 2D–F). The mean strength of correlation of the 4 significant associations identified was 0.457 ± 0.087, and LDA_1_ explained 90.0% ± 4.0% of the total variance. Postoperative performance was associated predominantly with contact location along a superioinferior (*z*) axis, as 50.5% ± 3.3% of LDA_1_ vector projected back onto the MNI z axis, whereas 20.5% ± 16.5% and 29.0% ± 13.5% projected on to the MNI x and y axes, respectively. Postoperative worsening was associated with more superior contact locations, while improvement was associated with more inferior contacts located near the center of the posterior (sensorimotor) STN (Fig. 2 and Table 2, S5).

**Table 2:** Neuropsychological tests administered and statistically significant results for entire cohort and subcohorts based on depression history.

Test	Primary domain	Secondary domain	Cohort	No Dep	+ Dep
RBANS DIGIT SPAN FORWARD	Attention				
WAIS DIGIT SPAN BACKWARD	Attention	Executive			
WAIS PICTURE COMPLETION	Attention	Visuospatial			R, L
COLOR-WORD INTERFERENCE TEST (STROOP ANALOG FROM D-KEFS)	Executive				R
TRAIL MAKING TEST-B	Executive				
WASI-II MATRIX REASONING	Executive	Visuospatial			R
WASI-II SIMILARITIES	Executive	Language			
CONTROLLED ORAL-WORD ASSOCIATION TEST (F-A-S)	Language	Executive			R, L
BOSTON NAMING TEST	Language	Visuospatial			
RBANS SEMANTIC FLUENCY	Language		L	L	R
WECHSLER TEST OF ADULT READING (WTAR)	Language				
RBANS LIST LEARNING	Memory		L	L	
RBANS LIST RECALL	Memory		R, L		R
RBANS FIGURE RECALL	Memory	Visuospatial			
RBANS FIGURE COPY	Visuospatial				R, L
ZUNG SELF-RATING ANXIETY SCALE (SAS)	Psychiatric				R, L
CENTER FOR EPIDEMIOLOGIC STUDIES DEPRESSION SCALE (CES-D)	Psychiatric				R
APATHY EVALUATION SCALE	Psychiatric				R

Secondary cognitive domain only listed if applicable. ‘L’ and ‘R’ indicate statistically significant Pearson correlation between active contact location in the left (L) or right (R) hemisphere and change in postoperative test result.

### Effect of psychiatric history and lead location on outcomes

**Fig. 3:**
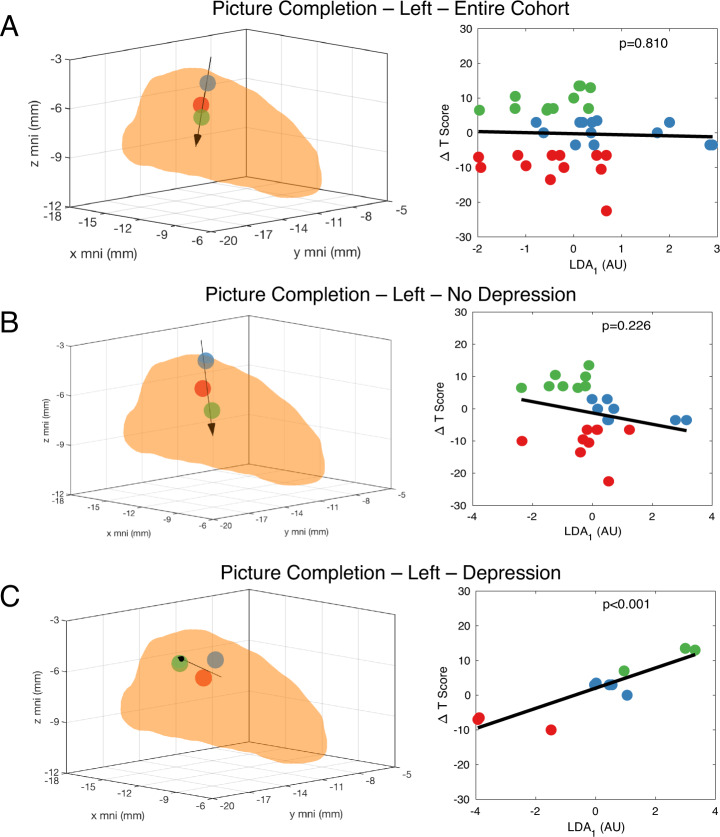
History of depression can moderate relationships between active contact location and post-DBS cognitive change. LDA analysis does not identify a significant linear relationship between active contact location and postoperative performance (postoperative worsening—red; no postoperative change—blue; postoperative improvement—green) on the picture completion assessment for the entire cohort (**A**) or for the subgroup of individuals without a history of depression (**B**). However, a statistically significant linear relationship is detected in the subgroup of patients with a history of depression (**C**).

Next, we divided the cohort into those with and without a history of depression, anxiety, and VH and repeated the same analyses. Figure 3 illustrates this approach with the picture completion task (attention and visuospatial measure). First, the result from the whole cohort analysis described previously is shown (Fig. 3A), with no meaningful correlation detected. Then, the cohort was split into those patients without and with a history of depression, respectively (Fig. 3B, C). While no correlation exists for those without a history of depression, we observed a strong linear correlation for those with a history of depression between improved postoperative performance and more posterior STN stimulation.

In similar fashion we ultimately detected 14 significant correlations for patients with a history of depression. We found more significant associations in the right hemisphere, which also had more overall variability in targeting (compare Fig. 1C, D). The mean strength of correlation was 0.700 ± 0.072, and LDA_1_ explained 90.8% ± 10.4% of the total variance. Only one of these correlations overlapped with those identified for the entire cohort (list recall, right side) (Table 2, S6). In contrast, we found only 2 correlations for those without a history of depression, both of which previously had been identified in the whole cohort analysis (Table 2, S7).

**Fig. 4:**
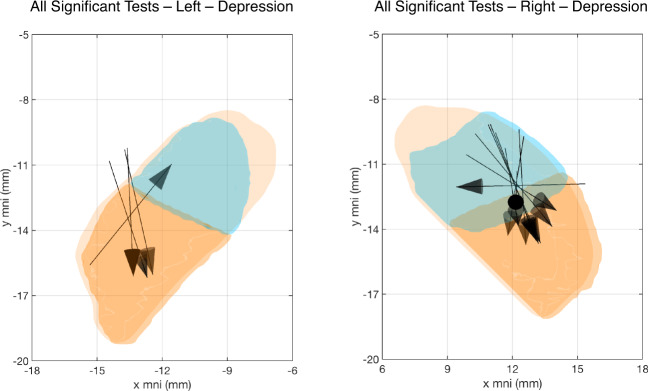
Variation along the anterior–posterior (*y*) axis primarily explains variation in postoperative performance in patients with a history of depression. Each vector identified by LDA and correlation analysis as representing a statistically significant linear relationship between contact location and postoperative performance on the respective cognitive test is superimposed on to one STN for each hemisphere for patients with a history of depression to illustrate the importance of the anterior-posterior vector component in this subgroup. For clarity, the figures have been simplified to 2 dimensions (*x* and *y*) only. The tripartite coloring of the STN reflects sensorimotor (orange), associative (turquoise), and limbic (tan) subregions according to the distal minimal atlas.

Whereas the predominant vector directions determined by LDA for both the whole cohort and the no depression history subgroup indicated improved outcomes associated primarily with more inferior stimulation locations (and poorer outcomes with more superior ones), the vectors for those with a history of depression instead revealed improved performance generally associated with more posterior stimulation locations (and poorer outcomes with more anterior ones) for 12 of the 14 significant correlations (Fig. 4, Tables S6, 7). For these 12, 53.1% ± 17.9% of the LDA_1_ vector projected onto the MNI y axis, whereas only 17.4% ± 13.1% and 29.5% ± 19.9% projected onto the MNI *x* and *z* axes, respectively. The two exceptions were the figure copy test for the right and left hemispheres, with improved performance associated with more medial and anteromedial stimulation, respectively.

We repeated our approach according to anxiety history. For those with a history of anxiety, we detected 6 significant correlations, 3 of which overlapped with those found for the entire cohort analysis (Tables S8, 9). The mean strength of correlation for these 6 was 0.720 ± 0.074, and LDA_1_ explained 90.9% ± 9.0% of the total variance. There was no predominant vector orientation (Fig. S2), with 20.6% ± 23.3%, 39.8% ± 28.7%, and 39.5% ± 30.2% of the LDA_1_ vector projecting onto the MNI x, y, and z axes, respectively.

For those without documented history of anxiety, we found 7 statistically significant correlations (Tables S8, S10). The mean strength of correlation for these 7 was 0.560 ± 0.091, and LDA_1_ explained 84.3% ± 15.4% of the total variance. Similar to analyses of the entire cohort and no history of depression subgroup, improved outcomes were associated primarily with more inferior stimulation locations (Fig. S2); 53.7% ± 16.6% of the LDA_1_ vector projected onto the MNI z axis, while 23.0% ± 14.6% and 23.2% ± 10.9% projected onto the x and y axes, respectively.

Next, we repeated our approach for patients with and without a history of VH but found no significant associations (data not shown).

### Volume of tissue activated analysis

We considered whether differences in stimulation intensity (i.e., total voltage or current) might explain our findings, but total VTAs between the subgroups were statistically indistinguishable (Table S11). Further, to complement our detailed analyses of active contact location, we also compared spatial distributions of stimulation by comparing the proportion of the VTA within the STN and its subregions (Table S11). History of anxiety was associated with a greater proportion of stimulation within the STN, but otherwise we found no differences.

Finally, we assessed for correlations between the ratio of motor to nonmotor STN stimulation as determined by VTA (Table S11) and change in NPT in the full cohort and subgroups. Only improved performance on semantic fluency in those with a history of anxiety with a greater ratio of motor to nonmotor stimulation of the left STN was statistically significant after correction for multiple comparisons (Table S12).

## Discussion

We used a supervised machine learning algorithm to investigate the relationships between location of stimulation within the STN and neuropsychological outcomes following DBS for PD in a retrospective cohort. We found a limited number of associations for the cohort as a whole, with most of the variance in postoperative change associated with location along a superioinferior (*z*) axis. Constraining the analysis to patients with a history of depression or anxiety revealed a substantially increased number of such associations. For patients with a history of depression, the variance in postoperative change was best explained by location along an anterioposterior (y) axis, suggesting these patients may be particularly sensitive to stimulation in more anterior STN regions. This is the first study, to our knowledge, to demonstrate an interaction between psychiatric history and location of STN stimulation on cognitive and psychiatric measures.

Research increasingly has been focused on uncovering relationships between STN stimulation location and both motor and non-motor outcomes [13–22, 46, 49–55]. We hypothesized that incorporation of a patient’s psychiatric history into such analyses might reveal new associations. Psychiatric symptoms like depression and anxiety can be among the first symptoms of PD, sometimes predating motor symptoms by years or decades [24, 25], and vulnerability to these symptoms may be a marker of underlying neurobiological differences. Furthermore, primary psychiatric disorders are associated with altered functional connectivity among neural networks [26–32], again raising the possibility of distinct effects of stimulation even in those patients with more long-standing psychiatric histories.

Supporting our hypothesis, division of the cohort according to history of depression revealed many more statistically significant associations in patients with a history of depression as compared to the overall cohort (14 vs 4; Table 2). Just as striking was the contrast in orientations of the three-dimensional vectors that best explained the variance in the data. For the whole cohort, those without a history of depression, and those without a history of anxiety, variation along the z (inferiosuperior) MNI axis best explained class differences, with improved nonmotor test results associated with more inferior stimulation located near the center of the sensorimotor subregion (Fig. 2, S2). In those with a history of depression, improved outcomes again generally were associated with central sensorimotor region stimulation, but the variance instead was best explained by location along the y (anterioposterior) MNI axis (Fig. 4). Interestingly, the calculated vectors pointed away from the more anterior limbic and associative STN subregions (Fig. 4), suggesting that patients with a history of depression may be uniquely sensitive to electrical fields induced in non-motor territories.

Analysis of LDA vector directions in those with a history of depression revealed one clear outlier: improved performance on the figure copy test was associated with more anteromedial (left STN) and medial (right STN) stimulation, respectively (Fig. 4, Table S6). The bilateral nature and shared importance of the mediolateral axis for this result argues against its being a false positive. Interestingly, the figure copy test was the only primary visuospatial assessment included.

There is ample evidence of a unique relationship between depression and PD. Depression is more common in the years prior to a PD diagnosis [24, 25], one of the most prevalent nonmotor symptoms of PD [56], and among the greatest contributors to overall quality of life [57]. The symptom profile of PD depression also may be partially distinct from that of idiopathic depression [58]. Finally, PD depression has been related to deficits in dopaminergic signaling from the ventral tegmental area to the ventral striatum (mesolimbic) and to the ventromedial prefrontal cortex (mesocortical) [59–63]. Notably, the ventromedial prefrontal cortex projects to the anterior STN [11].

In contrast to depression, results for anxiety were less clear-cut. Analysis of patients with and without a history of anxiety did reveal a greater number of significant associations in both sub-groups as compared to the cohort as a whole despite smaller sample sizes, consistent with some underlying significance to the diagnosis. However, there was no consistent orientation of the vectors best explaining variance in the data for those with a history of anxiety (Fig. S2), whereas the predominant source of variance for the no anxiety history group was along the superioinferior (z) axis, similar to the whole cohort. Taken together, these results suggest that more heterogeneous factors underlie the detected associations. For example, the anxiety experienced as dopaminergic medications wear off (‘off anxiety’) [64] might intermix with other forms, thereby producing less consistent results.

Alternatively, since depression and anxiety are often comorbid–although dissociable [65]–in PD, these findings could be influenced by those with a comorbid history of depression. This was a minority of patients in the history of anxiety subgroup (5 of 15), however, and there was only minimal overlap of identified location-outcome associations (2 neuropsychological tests, compare Table 2 to Table S8) between the history of depression and history of anxiety subgroups. Similarly, influential vector orientations were distinct between the two subgroups. Thus, there is little support for this explanation.

We previously found that a history of VH is associated with declines in attention and executive function following DBS [66], but no significant associations between postoperative testing changes and active contact location for those with a history of VH was elucidated in the current study. As controlling for the preoperative burden of depression and anxiety altered the statistically significant postoperative NPT changes identified only in this subgroup (Tables S2–4), any location-outcome relationships may be substantially moderated by comorbidities or the more widespread cortical Lewy body burden in this population [67] such that a different analysis approach is required.

Interestingly, our approach of using LDA to assess for relationships between active contact location and NPT changes was more sensitive than our analyses that utilized VTA despite only the latter incorporating stimulation intensity. The ratio of motor to nonmotor STN VTA previously has been related to neuropsychiatric changes after DBS [68], but we detected only one significant correlation with this approach. There was considerable variability in the VTA data (note large SDs in Table S11), which may explain why statistically significant differences were few, or perhaps the motor to nonmotor ratio is not the ideal metric. Furthermore, although lead localizations themselves are subject to some degree of error, they are more directly validated (for example, see Nowacki et al., 2018 [69]) than the models used to calculate VTAs. Our study included different electrode types (using constant voltage or constant current modes, respectively) and different modes of stimulation (monopolar and bipolar), both of which may impact VTA model accuracy. Additionally, the VTA model is binary, simplifying stimulation to either activating or not activating axons within individual voxels, which may not account for effects from stimulation of other local cellular components [70]. In fact, recent work suggests that estimates of electric field magnitude may explain variance in outcome data more effectively than VTA [42], which has led to greater incorporation of other such approaches in newer work [22, 71]. In our case, future analyses of possible differences in connectivity of stimulation sites in our subgroups with other brain regions might enhance the utility of these VTA calculations.

Beyond the aforementioned concerns related to lead localization and VTA model accuracy, we acknowledge several additional limitations to our approach. The retrospective design was dependent on chart review for psychiatric history, and without a structured interview by a trained psychiatrist, we may have missed relevant history. Given the conceptual framework that motivated our hypothesis, we focused on any history of psychiatric diagnosis and not just preoperative symptoms. Patients with active mood symptoms may be excluded from DBS surgery [72], and, consistent with this, our subgroups of those with a history of depression, anxiety, or VH scored similarly to those without on preoperative NPT measures of depression, anxiety, and apathy. This argues against the presence of active psychiatric symptoms at baseline as an explanation for our findings. Our study did not include motor outcomes, as quantification of such changes (i.e., UPDRS scores) was not routinely performed postoperatively at our institution—others have reported that motor and cognitive outcomes are not correlated [73]. We also only had limited measures available of potential mood elevation or impulsivity that may be induced by nonmotor STN stimulation [18, 21], though this concern is mitigated by the longer-term nature of our follow-up (mean time until follow-up of 1.4 years) and the resulting likelihood that any clinically obvious hypomania or impulsivity would have been managed with changes to stimulation parameters prior to acquisition of follow-up NPT. That said, the longer-term follow-up might also be considered a limitation, as there is more time for progressive neurodegeneration, medication changes, and other such changes to affect results independent of chronic stimulation. Finally, approximately 75% of STN-DBS patients in our database did not complete postoperative NPT, which may bias the data in unknown fashion.

In conclusion, PD patients with a history of depression may be especially sensitive to off-target stimulation of non-motor STN subregions. Our results support efforts to take extra care during surgical targeting in this population. For instance, staged lead implantation to reduce the chance of brain shift affecting the contralateral side could be considered, as the greater variability in targeting of the right STN in our cohort is likely related to the left STN always being implanted first in this cohort. More broadly, incorporating presurgical psychiatric history into surgical target planning may be an important step towards improving outcomes, and prospective studies are needed to validate our findings.

## Supplementary information

Figure S1

Figure S2

Supplementary Figure Legends

Supplementary Tables

## Data Availability

Computer code used for LDA is available via the corresponding author.
