# Supplementary material for: Depression history modulates effects of subthalamic nucleus topography on neuropsychological outcomes of deep brain stimulation for Parkinson’s disease
Source: Transl Psychiatry. 2022 May 27;12:213. doi: 10.1038/s41398-022-01978-y (PMC9142573; doi:10.1038/s41398-022-01978-y)
Supplement: Supplementary file 1 — Figure S1 [file 41398_2022_1978_MOESM1_ESM.pdf]

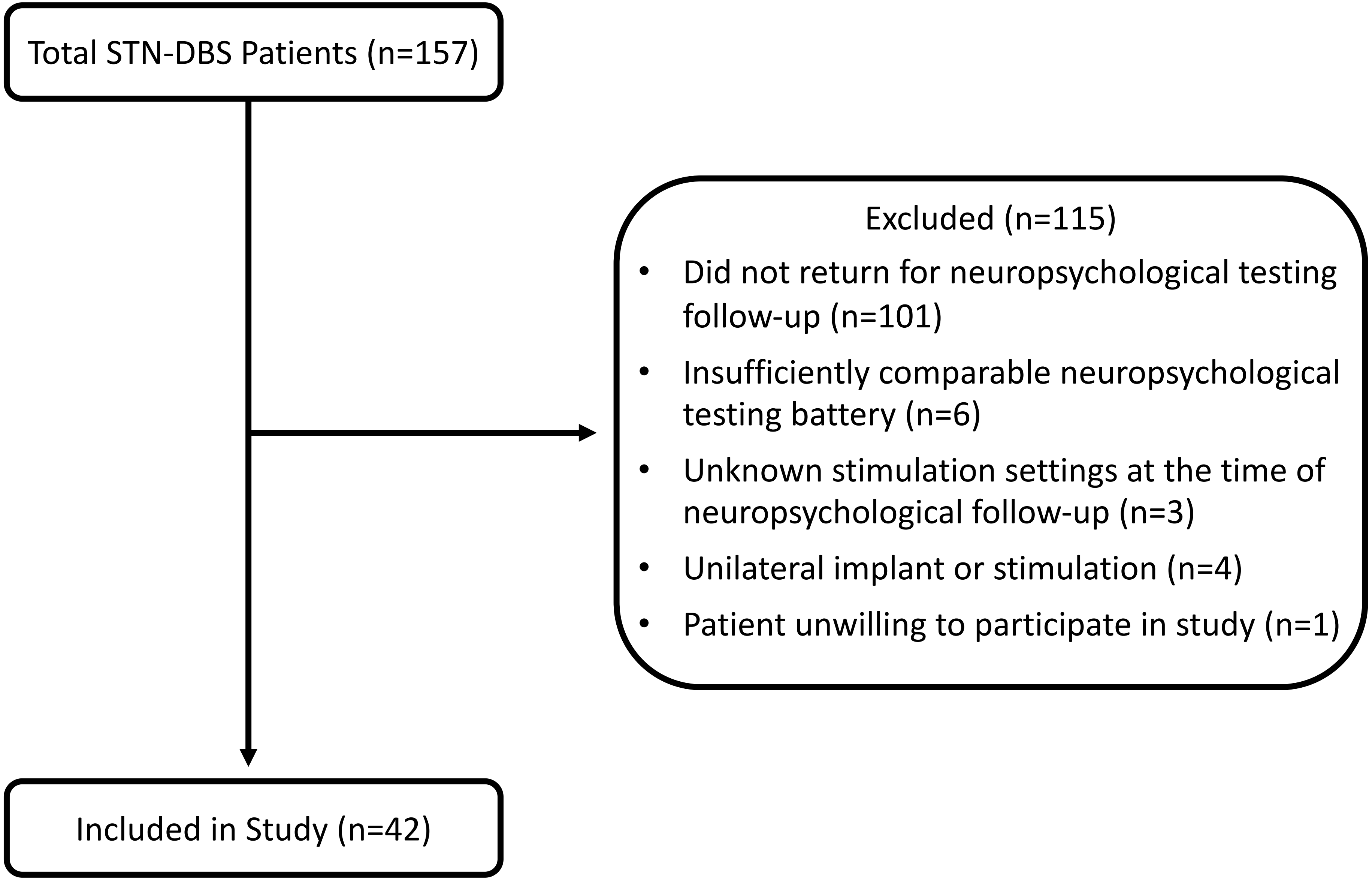
Total STN-DBS Patients (n=157)
Excluded (n=115)
• Did not return for neuropsychological testing follow-up (n=101)
• Insufficiently comparable neuropsychological testing battery (n=6)
• Unknown stimulation settings at the time of neuropsychological follow-up (n=3)
• Unilateral implant or stimulation (n=4)
• Patient unwilling to participate in study (n=1)
Included in Study (n=42)
