# Supplementary figures and images for: Depression history modulates effects of subthalamic nucleus topography on neuropsychological outcomes of deep brain stimulation for Parkinson’s disease
Source: Transl Psychiatry. 2022 May 27;12:213. doi: 10.1038/s41398-022-01978-y (PMC9142573; doi:10.1038/s41398-022-01978-y)

Anxiety

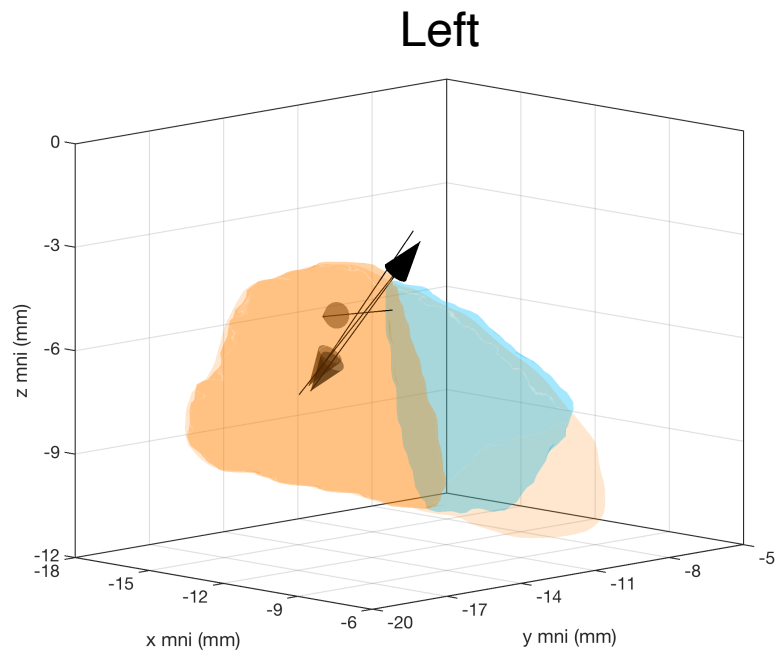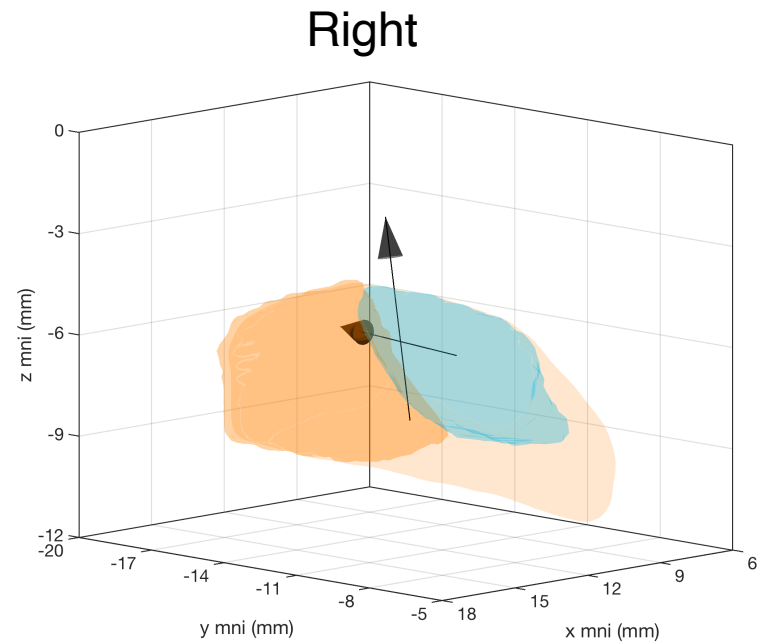

No Anxiety

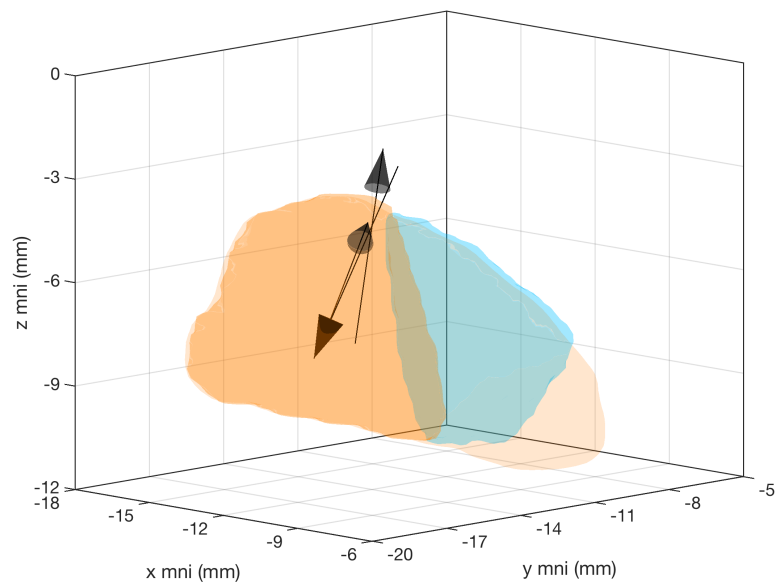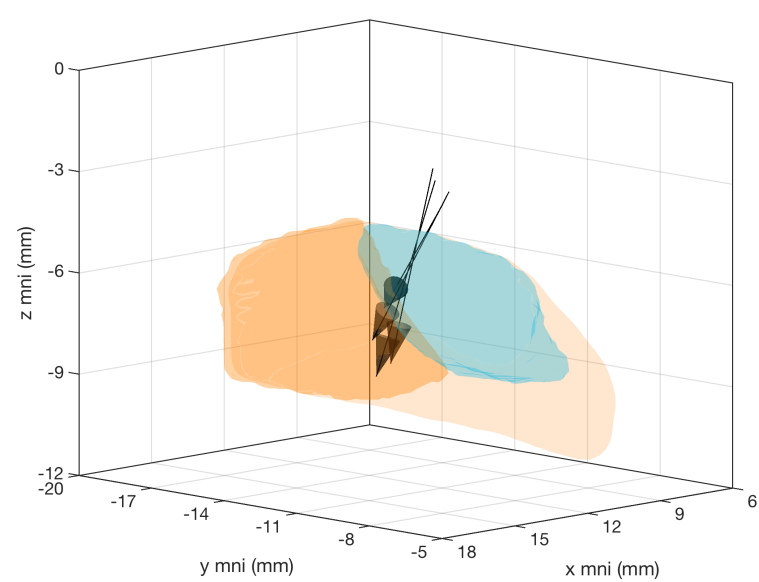

Supplement: Supplementary file 2 — Figure S2 [file 41398_2022_1978_MOESM2_ESM.pdf]
