# Supplementary material for: Depression history modulates effects of subthalamic nucleus topography on neuropsychological outcomes of deep brain stimulation for Parkinson’s disease
Source: Transl Psychiatry. 2022 May 27;12:213. doi: 10.1038/s41398-022-01978-y (PMC9142573; doi:10.1038/s41398-022-01978-y)
Supplement: Supplementary file 3 — Supplementary Figure Legends [file 41398_2022_1978_MOESM3_ESM.docx]

**SUPPLEMENTARY FIGURE LEGENDS**

**Figure S1. CONSORT-style diagram of study.**

**Figure S2. Significant LDA vectors in patients with and without a history of anxiety.** Each vector identified by LDA and correlation analysis as representing a statistically significant linear relationship between contact location and postoperative performance on the respective cognitive test is superimposed on to one STN for each hemisphere for patients with and without a history of anxiety to illustrate the lack of cohesive vector directions in those with a history of anxiety. The tripartite coloring of the STN reflects sensorimotor (orange), associative (turquoise), and limbic (tan) subregions according to the distal minimal atlas.
