# Supplementary material for: Depression history modulates effects of subthalamic nucleus topography on neuropsychological outcomes of deep brain stimulation for Parkinson’s disease
Source: Transl Psychiatry. 2022 May 27;12:213. doi: 10.1038/s41398-022-01978-y (PMC9142573; doi:10.1038/s41398-022-01978-y)
Supplement: Supplementary file 4 — Supplementary Tables [file 41398_2022_1978_MOESM4_ESM.docx]

**Table S1. Baseline Neuropsychology Testing by Psychiatric History**

| TEST | ALL | DEPRESSION | ANXIETY | VH |
| --- | --- | --- | --- | --- |
| RBANS Digit Span Forward | 52.6 (10.5) | 47.9 (9.0) | 51.9 (9.6) | 51.5 (11.1) |
| WAIS Digit Span Backward | 52.2 (7.8) | 50.2 (6.5) | 49.4 (7.2) | 55.3 (8.5) |
| WAIS Picture Completion | 49.7 (8.4) | 46.0 (5.7) | 49.0 (8.3) | 48.3 (6.0) |
| Color-Word Interference Test  (Stroop analogue) | 50.5 (9.2) | 49.6 (8.7) | 50.1 (11.1) | 52.3 (12.6) |
| Trail Making Test-B | 46.6 (18.7) | 47.0 (12.3) | 37.9 (26.8) | 46.0 (18.4) |
| WASI-II Matrix Reasoning | 50.7 (10.9) | 48.1 (10.9) | 47.9 (11.1) | 50.6 (10.8) |
| WASI-II Similarities | 52.8 (10.3) | 51.3 (11.7) | 51.5 (10.0) | 51.6 (9.3) |
| Controlled Oral-Word Association  test (F-A-S) | 50.0 (9.7) | 43.7 (8.1)** | 50.0 (9.7) | 48.9 (6.6) |
| Boston Naming Test | 55.4 (9.2) | 53.6 (12.5) | 54.1 (8.0) | 53.9 (12.6) |
| RBANS Semantic Fluency | 45.0 (7.5) | 41.0 (7.2)* | 43.2 (6.5) | 44.1 (7.6) |
| Wechsler Test of Adult Reading  (WTAR) | 54.6 (8.4) | 56.0 (9.8) | 54.6 (9.2) | 53.1 (9.7) |
| RBANS List Learning | 43.4 (10.2) | 36.6 (10.8)** | 42.0 (7.3) | 43.2 (7.5) |
| RBANS List Recall | 46.8 (8.7) | 45.6 (10.7) | 45.3 (9.1) | 46.8 (9.0) |
| RBANS Figure Recall | 48.5 (10.9) | 45.0 (12.8) | 45.7 (11.8) | 44.5 (13.0) |
| RBANS Figure Copy | 48.5 (15.9) | 42.7 (22.3) | 45.0 (18.9) | 40.8 (21.5) |
| Zung Self-Rating Anxiety scale (SAS) | 42.3 (8.9) | 43.9 (9.2) | 42.6 (10.6) | 41.8 (8.9) |
| center for epidemiologic studies  depression scale (CES-D) | 12.7 (9.6) | 14.3 (10.1) | 16.9 (11.2) | 14.9 (11.7) |
| Apathy Evaluation scale^‡^ | 26.5 (10.5) | 28.6 (13.0) | 26.5 (11.6) | 27.7 (12.1) |

All values represent mean (SD) T-scores, with the exceptions of SAS (presented as the SAS anxiety index) and CES-D and apathy evaluation (presented as raw scores), respectively. Statistical comparisons are made between yes/no condition for depression, anxiety, and VH. Note that data for ‘no’ conditions are not shown for clarity. Instead, mean numbers for the entire cohort are also shown without any further statistical analysis. VH = visual hallucinations. ^‡^ = scoring range of 18-72, with higher values representing greater apathy. *p<0.05, **p<0.01.

**Table S2. Postoperative Neuropsychology Testing Changes**

| TEST | ALL | DEPRESSION | ANXIETY | VH |
| --- | --- | --- | --- | --- |
| RBANS Digit Span Forward | 0.9 (9.6) | 3.3 (8.2) | 1.1 (11.0) | -2.7 (12.0) |
| WAIS Digit Span Backward | -0.9 (9.4) | 0.0 (7.6) | 3.6 (10.2) | -3.7 (6.8) |
| WAIS Picture Completion | -0.3 (8.4) | 2.1 (7.7) | -3.1 (9.2) | -2.1 (8.0) |
| Color-Word Interference Test  (Stroop analogue) | -5.2 (11.6)* | -2.7 (12.4) | -1.2 (10.8) | -9.6 (15.1) |
| Trail Making Test-B | -8.4 (15.9)** | -8.4 (9.3)* | -7.3 (19.9) | -15.0 (21.6)* |
| WASI-II Matrix Reasoning | -1.5 (9.5) | -0.2 (6.8) | 0.2 (10.0) | -8.2 (10.8) |
| WASI-II Similarities | -2.4 (8.7) | -1.9 (8.3) | -1.7 (8.8) | -3.7 (10.2) |
| Controlled Oral-Word Association  test (F-A-S) | -5.6 (6.8)*** | -3.2 (7.6) | -2.9 (7.0) | -6.1 (7.8)* |
| Boston Naming Test | 1.1 (6.6) | 1.3 (6.9) | -1.2 (7.1) | -2.6 (7.1) |
| RBANS Semantic Fluency | -4.3 (9.2)** | -0.3 (11.2) | -1.0 (10.4) | -4.6 (8.3) |
| Wechsler Test of Adult Reading  (WTAR) | -0.1 (4.3) | 0.3 (1.5) | 0.7 (2.5) | 2.1 (6.0) |
| RBANS List Learning | -1.7 (10.8) | 1.8 (11.6) | -3.5 (8.9) | -9.3 (9.8)* |
| RBANS List Recall | -1.7 (10.3) | -1.1 (11.0) | -2.5 (11.7) | -8.0 (10.7)* |
| RBANS Figure Recall | 1.8 (11.9) | 3.2 (12.9) | 3.6 (13.9) | -3.8 (14.9) |
| RBANS Figure Copy | 2.7 (14.2) | 6.1 (15.1) | 3.5 (17.6) | 5.9 (17.4) |
| Zung Self-Rating Anxiety scale (SAS) | -1.7 (12.5) | -1.5 (13.8) | -1.7 (15.3) | -1.1 (11.0) |
| center for epidemiologic studies  depression scale (CES-D) | -1.5 (9.1) | -0.3 (10.8) | -5.6 (8.3)* | -3.1 (8.2) |
| Apathy Evaluation scale | 2.1 (10.9) | 4.6 (10.7) | 0.2 (8.1) | 3.1 (10.3) |

All values represent mean (SD) ΔT-scores (T_postoperative_ – T_preoperative_), with the exceptions of SAS (presented as the SAS anxiety index) and CES-D and apathy evaluation (presented as raw scores), respectively. Note that a negative change in SAS, CES-D, and the apathy evaluation represent improvement (i.e., a reduction in symptoms). Statistical analysis was via paired t-tests of the null hypothesis that ΔT =0. VH = visual hallucinations. *p<0.05, **p<0.01, ***p<0.001.

**Table S3. Postoperative Neuropsychology Testing Changes While Controlling for Baseline Depression**

| TEST | ALL | DEPRESSION | ANXIETY | VH |
| --- | --- | --- | --- | --- |
| RBANS Digit Span Forward | 1.4 (1.6) | 2.9 (2.6) | 2.6 (2.7) | -2.8 (4.2) |
| WAIS Digit Span Backward | -1.0 (1.7) | -0.6 (2.3) | 4.2 (2.9) | -4.9 (2.3) |
| WAIS Picture Completion | -0.3 (1.4) | 2.0 (2.3) | -3.1 (2.5) | -2.1 (2.8) |
| Color-Word Interference Test  (Stroop analogue) | -5.3 (2.1)* | -2.6 (4.4) | -0.5 (2.9) | -10.3 (6.0) |
| Trail Making Test-B | -7.5 (2.4)** | -8.1 (3.1)* | -3.6 (3.9) | -11.1 (6.8) |
| WASI-II Matrix Reasoning | -1.7 (1.6) | -0.3 (2.3) | 0.2 (2.8) | -9.4 (3.9)* |
| WASI-II Similarities | -2.6 (1.5) | -2.4 (2.7) | -1.7 (2.4) | -4.5 (3.8) |
| Controlled Oral-Word Association  test (F-A-S) | -5.2 (1.1)*** | -3.3 (2.4) | -2.3 (1.8) | -6.0 (3.0) |
| Boston Naming Test | 1.2 (1.0) | 0.4 (1.9) | -0.3 (1.7) | -3.1 (1.6) |
| RBANS Semantic Fluency | -3.2 (1.5)* | 0.3 (3.5) | -0.6 (2.8) | -2.5 (3.0) |
| Wechsler Test of Adult Reading  (WTAR) | 0.0 (0.7) | 0.3 (0.5) | 0.8 (0.7) | 2.4 (2.2) |
| RBANS List Learning | -1.4 (1.7) | 1.8 (3.5) | -2.3 (2.1) | -7.0 (3.4) |
| RBANS List Recall | -1.3 (1.6) | -1.1 (3.3) | -2.0 (3.2) | -5.7 (3.5) |
| RBANS Figure Recall | 2.2 (1.9) | 3.2 (3.9) | 5.4 (3.3) | -2.8 (5.2) |
| RBANS Figure Copy | 3.7 (2.4) | 6.1 (4.5) | 4.6 (4.7) | 8.9 (7.0) |
| Zung Self-Rating Anxiety scale (SAS) | -1.4 (2.1) | -1.5 (4.2) | -1.8 (4.2) | -1.3 (4.1) |
| Apathy Evaluation scale | 2.1 (2.1) | 4.6 (3.6) | 0.2 (2.3) | 3.1 (3.9) |

All values represent the linear regression estimate (SE) of ΔT-scores (T_postoperative_ – T_preoperative_), with the exceptions of SAS (presented as the SAS anxiety index) and CES-D (presented as a raw score), respectively. Note that a negative change in SAS and the apathy evaluation represent improvement (i.e., a reduction in symptoms). Statistical analysis was via generalized linear model controlling for baseline depression as measured by the CES-D with null hypothesis that ΔT =0. VH = visual hallucinations. SE = standard error. *p<0.05, **p<0.01, ***p<0.001.

**Table S4. Postoperative Neuropsychology Testing Changes While Controlling for Baseline Anxiety**

| TEST | ALL | DEPRESSION | ANXIETY | VH |
| --- | --- | --- | --- | --- |
| RBANS Digit Span Forward | 0.6 (1.6) | 2.9 (2.6) | 1.1 (2.9) | -2.8 (4.2) |
| WAIS Digit Span Backward | -1.4 (1.6) | -0.6 (2.3) | 3.6 (2.7) | -4.9 (2.3) |
| WAIS Picture Completion | -0.2 (1.4) | 2.0 (2.3) | -3.1 (2.5) | -2.1 (2.8) |
| Color-Word Interference Test  (Stroop analogue) | -5.2 (2.0)* | -2.6 (4.4) | -1.2 (2.8) | -10.3 (6.0) |
| Trail Making Test-B | -8.8 (2.7)** | -8.1 (3.1)* | -7.3 (5.1) | -11.1 (6.8) |
| WASI-II Matrix Reasoning | -2.1 (1.6) | -0.3 (2.3) | 0.2 (2.8) | -9.4 (3.9)* |
| WASI-II Similarities | -2.7 (1.5) | -2.4 (2.7) | -1.7 (2.4) | -4.5 (3.8) |
| Controlled Oral-Word Association  test (F-A-S) | -5.4 (1.1)*** | -3.3 (2.4) | -2.9 (1.8) | -6.0 (3.0) |
| Boston Naming Test | 1.0 (1.1) | 0.4 (1.9) | -1.2 (1.8) | -3.1 (1.6) |
| RBANS Semantic Fluency | -3.5 (1.5)* | 0.3 (3.5) | -1.0 (2.7) | -2.5 (3.0) |
| Wechsler Test of Adult Reading  (WTAR) | -0.1 (0.7) | 0.3 (0.5) | 0.7 (0.6) | 2.4 (2.2) |
| RBANS List Learning | -1.4 (1.7) | 1.8 (3.5) | -3.5 (2.3) | -7.0 (3.4) |
| RBANS List Recall | -1.3 (1.6) | -1.1 (33) | -2.5 (3.0) | -5.7 (3.5) |
| RBANS Figure Recall | 1.2 (2.0) | 3.2 (3.9) | 3.6 (3.6) | -2.8 (5.2) |
| RBANS Figure Copy | 3.4 (2.4) | 6.1 (4.5) | 3.5 (4.6) | 8.9 (7.0) |
| center for epidemiologic studies  depression scale (CES-D) | -1.7 (1.5) | -0.3 (3.2) | -5.6 (2.2)* | -3.1 (2.9) |
| Apathy Evaluation scale | 2.3 (2.1) | 4.6 (3.6) | 0.2 (2.3) | 3.1 (3.9) |

All values represent the linear regression estimate (SE) of ΔT-scores (T_postoperative_ – T_preoperative_), with the exceptions of CES-D and apathy evaluation (both presented as raw scores). Note that a negative change in CES-D and the apathy evaluation represent improvement (i.e., a reduction in symptoms). Statistical analysis was via generalized linear model controlling for baseline anxiety as measured by the SAS with null hypothesis that ΔT =0. VH = visual hallucinations. SE = standard error. *p<0.05, **p<0.01, ***p<0.001.

**Table S5. LDA and Correlation Results for Entire Cohort.**

| TEST | hemi | LDA_1_ prop | *r* | p | LDA_1_ %X | LDA_1_ %Y | LDA_1_ %Z |
| --- | --- | --- | --- | --- | --- | --- | --- |
| RBANS Digit Span Forward | Right | 0.54 | 0.10 | 0.53 | -32.22 | -24.06 | -43.72 |
| WAIS Digit Span Backward | Right | 0.66 | 0.21 | 0.20 | -9.04 | -79.47 | 11.49 |
| WAIS Picture Completion | Right | 0.95 | 0.22 | 0.20 | 28.69 | -51.38 | 19.93 |
| Stroop analogue | Right | 0.83 | 0.27 | 0.11 | 8.69 | -10.96 | 80.35 |
| Trail Making Test-B | Right | 0.97 | 0.24 | 0.13 | 67.65 | 22.37 | 9.98 |
| WASI-II Matrix Reasoning | Right | 0.74 | 0.17 | 0.31 | -19.66 | -32.28 | -48.06 |
| WASI-II Similarities | Right | 0.79 | 0.33 | 0.05 | 30.84 | -18.06 | -51.10 |
| F-A-S | Right | 0.89 | 0.35 | 0.03 | 4.18 | -30.46 | 65.36 |
| Boston Naming Test | Right | 0.93 | 0.18 | 0.26 | -30.69 | -33.55 | -35.76 |
| RBANS Semantic Fluency | Right | 0.61 | 0.20 | 0.21 | 40.87 | 18.97 | 40.16 |
| WTAR | Right | 0.92 | 0.20 | 0.23 | 56.10 | 36.16 | 7.74 |
| RBANS List Learning | Right | 0.89 | 0.26 | 0.10 | 36.82 | 22.85 | -40.33 |
| RBANS List Recall | Right | 0.92 | 0.41 | 0.01***** | 17.27 | -30.07 | -52.66 |
| RBANS Figure Recall | Right | 1.00 | 0.09 | 0.56 | -23.33 | -53.45 | -23.22 |
| RBANS Figure Copy | Right | 0.76 | 0.21 | 0.20 | -19.54 | 31.89 | -48.57 |
| SAS | Right | 0.84 | 0.32 | 0.05 | -23.60 | -73.70 | -2.69 |
| CES-D | Right | 0.94 | 0.19 | 0.26 | -12.56 | -42.24 | -45.19 |
| Apathy Evaluation scale | Right | 0.97 | 0.11 | 0.58 | -2.85 | 8.79 | 88.36 |
| RBANS Digit Span Forward | Left | 0.73 | 0.12 | 0.44 | 15.71 | -78.95 | -5.34 |
| WAIS Digit Span Backward | Left | 0.66 | 0.34 | 0.04 | -19.27 | 39.51 | 41.22 |
| WAIS Picture Completion | Left | 0.62 | 0.04 | 0.81 | 17.71 | -31.20 | -51.09 |
| Stroop analogue | Left | 0.79 | 0.09 | 0.59 | 25.90 | 35.14 | 38.96 |
| Trail Making Test-B | Left | 0.84 | 0.16 | 0.30 | -51.76 | 30.02 | 18.22 |
| WASI-II Matrix Reasoning | Left | 0.99 | 0.22 | 0.18 | 0.53 | 10.25 | 89.21 |
| WASI-II Similarities | Left | 0.90 | 0.08 | 0.65 | 10.25 | 58.91 | 30.84 |
| F-A-S | Left | 0.55 | 0.31 | 0.05 | 32.59 | -50.16 | -17.25 |
| Boston Naming Test | Left | 0.81 | 0.25 | 0.12 | 21.42 | -22.72 | -55.86 |
| RBANS Semantic Fluency | Left | 0.92 | 0.44 | <0.01***** | 7.80 | -39.14 | -53.06 |
| WTAR | Left | 0.95 | 0.36 | 0.03 | 26.28 | -40.87 | -32.85 |
| RBANS List Learning | Left | 0.84 | 0.40 | 0.01***** | -44.47 | 9.60 | -45.94 |
| RBANS List Recall | Left | 0.92 | 0.59 | <0.0001***** | -12.35 | -37.16 | -50.49 |
| RBANS Figure Recall | Left | 0.79 | 0.05 | 0.74 | -0.03 | 87.04 | 12.94 |
| RBANS Figure Copy | Left | 0.97 | 0.06 | 0.71 | 39.06 | 39.70 | 21.24 |
| SAS | Left | 0.92 | 0.36 | 0.03 | 21.40 | -66.62 | 11.99 |
| CES-D | Left | 0.82 | 0.13 | 0.45 | 74.58 | -18.16 | 7.25 |
| Apathy Evaluation scale | Left | 0.75 | 0.01 | 0.98 | -45.70 | 13.33 | 40.97 |

Hemi = hemisphere. LDA_1_ Prop = proportion of the total variance explained by the principle axis of variation as determined by LDA. *r* = Pearson’s *r*. p = unadjusted p-value. LDA_1_% refers to the percentage of the principle LDA axis that corresponds to each traditional MNI axis (X, Y, and Z) with sign indicating directionality. Note that per MNI convention, a negative X component indicates the medial direction for the right hemisphere but the lateral direction for the left. ***** = p-value is significant after false discovery rate correction.

**Table S6. LDA and Correlation Results for the History of Depression Subcohort.**

| TEST | hemi | LDA_1_ prop | *r* | p | LDA_1_ %X | LDA_1_ %Y | LDA_1_ %Z |
| --- | --- | --- | --- | --- | --- | --- | --- |
| RBANS Digit Span Forward | Right | 0.96 | 0.15 | 0.65 | 6.42 | 53.34 | 40.24 |
| WAIS Digit Span Backward | Right | 0.92 | 0.13 | 0.68 | -28.40 | 27.81 | -43.79 |
| WAIS Picture Completion | Right | 0.95 | 0.62 | 0.04***** | 19.50 | -51.85 | 28.65 |
| Stroop analogue | Right | 1.00 | 0.67 | 0.03***** | 40.82 | -25.31 | 33.87 |
| Trail Making Test-B | Right | 0.77 | 0.19 | 0.58 | 34.09 | 50.60 | 15.31 |
| WASI-II Matrix Reasoning | Right | 0.90 | 0.74 | 0.01***** | 3.84 | -63.37 | -32.79 |
| WASI-II Similarities | Right | 0.89 | 0.38 | 0.25 | 31.23 | -15.44 | 53.33 |
| F-A-S | Right | 0.85 | 0.68 | 0.01***** | 6.80 | -39.83 | 53.37 |
| Boston Naming Test | Right | 0.97 | 0.40 | 0.19 | -29.39 | -55.37 | -15.24 |
| RBANS Semantic Fluency | Right | 0.96 | 0.61 | 0.04***** | 35.15 | -42.47 | 22.38 |
| WTAR | Right | N/A | N/A | N/A | N/A | N/A | N/A |
| RBANS List Learning | Right | 0.85 | 0.42 | 0.20 | -48.92 | -17.52 | 33.56 |
| RBANS List Recall | Right | 1.00 | 0.64 | 0.03***** | 28.38 | -64.25 | 7.37 |
| RBANS Figure Recall | Right | 0.96 | 0.29 | 0.38 | -52.72 | 3.45 | -43.83 |
| RBANS Figure Copy | Right | 0.98 | 0.76 | 0.01***** | -86.84 | -2.08 | 11.09 |
| SAS | Right | 0.96 | 0.73 | 0.01***** | 25.85 | -64.02 | 10.12 |
| CES-D | Right | 0.95 | 0.72 | 0.01***** | -8.10 | -50.23 | -41.67 |
| Apathy Evaluation scale | Right | 0.76 | 0.70 | 0.04***** | -0.96 | -23.65 | 75.39 |
| RBANS Digit Span Forward | Left | 0.80 | 0.20 | 0.54 | -11.16 | -56.28 | 32.56 |
| WAIS Digit Span Backward | Left | 0.98 | 0.51 | 0.09 | 4.32 | 50.61 | 45.06 |
| WAIS Picture Completion | Left | 0.95 | 0.89 | <0.001***** | 19.01 | -58.44 | 22.55 |
| Stroop analogue | Left | 0.98 | 0.51 | 0.14 | 25.50 | -9.04 | 65.46 |
| Trail Making Test-B | Left | 0.99 | 0.28 | 0.40 | 47.12 | -36.05 | -16.83 |
| WASI-II Matrix Reasoning | Left | 0.93 | 0.56 | 0.07 | 12.24 | -76.36 | 11.41 |
| WASI-II Similarities | Left | 0.90 | 0.55 | 0.08 | 30.70 | -58.17 | -11.13 |
| F-A-S | Left | 0.62 | 0.65 | 0.02***** | 16.02 | -70.66 | -13.32 |
| Boston Naming Test | Left | 0.77 | 0.41 | 0.18 | 7.79 | 78.12 | -14.08 |
| RBANS Semantic Fluency | Left | 0.74 | 0.27 | 0.40 | 6.28 | -39.94 | -53.78 |
| WTAR | Left | N/A | N/A | N/A | N/A | N/A | N/A |
| RBANS List Learning | Left | 0.91 | 0.49 | 0.13 | -60.02 | -18.37 | -21.61 |
| RBANS List Recall | Left | 0.74 | 0.54 | 0.09 | -0.38 | -83.57 | -16.05 |
| RBANS Figure Recall | Left | 0.64 | 0.18 | 0.59 | -14.35 | 85.58 | -0.07 |
| RBANS Figure Copy | Left | 0.92 | 0.66 | 0.03***** | 43.23 | 53.25 | 3.52 |
| SAS | Left | 0.91 | 0.69 | 0.02***** | 3.96 | -82.97 | -13.07 |
| CES-D | Left | 0.97 | 0.14 | 0.68 | 29.57 | 37.34 | 33.09 |
| Apathy Evaluation scale | Left | 0.71 | 0.53 | 0.14 | -38.37 | 57.13 | 4.50 |

Hemi = hemisphere. LDA_1_ Prop = proportion of the total variance explained by the principle axis of variation as determined by LDA. *r* = Pearson’s *r*. p = unadjusted p-value. LDA_1_% refers to the percentage of the principle LDA axis that corresponds to each traditional MNI axis (X, Y, and Z) with sign indicating directionality. Note that per MNI convention, a negative X component indicates the medial direction for the right hemisphere but the lateral direction for the left. N/A = LDA unable to be run due to insufficient data range to establish three distinct performance classes as described in the methods section. ***** = p-value is significant after false discovery rate correction.

**Table S7. LDA and Correlation Results for the No History of Depression Subcohort.**

| TEST | hemi | LDA_1_ prop | *r* | p | LDA_1_ %X | LDA_1_ %Y | LDA_1_ %Z |
| --- | --- | --- | --- | --- | --- | --- | --- |
| RBANS Digit Span Forward | Right | 0.56 | 0.32 | 0.09 | -46.86 | -41.27 | -11.87 |
| WAIS Digit Span Backward | Right | 0.64 | 0.38 | 0.05 | -29.41 | -57.64 | -12.96 |
| WAIS Picture Completion | Right | 0.92 | 0.15 | 0.47 | 39.29 | -39.34 | 21.37 |
| Stroop analogue | Right | 1.00 | 0.44 | 0.02 | 12.89 | 11.32 | -75.79 |
| Trail Making Test-B | Right | 0.96 | 0.29 | 0.12 | 84.72 | 14.13 | 1.14 |
| WASI-II Matrix Reasoning | Right | 0.76 | 0.27 | 0.19 | -21.87 | 8.64 | -69.49 |
| WASI-II Similarities | Right | 0.84 | 0.46 | 0.02 | 10.54 | -6.31 | -83.15 |
| F-A-S | Right | 0.89 | 0.16 | 0.43 | 4.37 | -45.31 | 50.32 |
| Boston Naming Test | Right | 0.99 | 0.36 | 0.06 | -30.46 | -37.90 | -31.64 |
| RBANS Semantic Fluency | Right | 0.87 | 0.26 | 0.17 | -8.23 | -35.73 | -56.04 |
| WTAR | Right | 0.91 | 0.25 | 0.19 | 63.75 | 28.50 | -7.76 |
| RBANS List Learning | Right | 0.96 | 0.21 | 0.27 | 33.82 | 17.75 | -48.43 |
| RBANS List Recall | Right | 0.94 | 0.41 | 0.02 | 11.04 | -15.03 | -73.93 |
| RBANS Figure Recall | Right | 0.91 | 0.02 | 0.90 | -30.12 | -46.54 | -23.34 |
| RBANS Figure Copy | Right | 0.93 | 0.26 | 0.18 | -31.13 | 34.92 | -33.96 |
| SAS | Right | 0.93 | 0.26 | 0.20 | -64.80 | -27.79 | -7.41 |
| CES-D | Right | 0.97 | 0.02 | 0.92 | 14.83 | 40.28 | 44.89 |
| Apathy Evaluation scale | Right | 0.90 | 0.32 | 0.18 | -27.96 | 13.18 | -58.87 |
| RBANS Digit Span Forward | Left | 0.60 | 0.07 | 0.72 | 39.87 | -16.06 | -44.07 |
| WAIS Digit Span Backward | Left | 0.72 | 0.45 | 0.02 | -34.39 | 25.28 | 40.34 |
| WAIS Picture Completion | Left | 0.85 | 0.25 | 0.23 | 22.08 | -9.69 | -68.24 |
| Stroop analogue | Left | 0.84 | 0.18 | 0.37 | -6.52 | 10.76 | 82.72 |
| Trail Making Test-B | Left | 0.71 | 0.03 | 0.86 | -58.35 | 25.37 | 16.28 |
| WASI-II Matrix Reasoning | Left | 0.71 | 0.14 | 0.49 | 0.53 | -8.50 | -90.97 |
| WASI-II Similarities | Left | 0.75 | 0.06 | 0.78 | -17.37 | -74.50 | 8.13 |
| F-A-S | Left | 0.54 | 0.01 | 0.96 | 42.10 | -48.04 | -9.86 |
| Boston Naming Test | Left | 0.86 | 0.40 | 0.03 | 18.55 | -33.29 | -48.16 |
| RBANS Semantic Fluency | Left | 0.96 | 0.50 | 0.01***** | 8.59 | -34.20 | -57.21 |
| WTAR | Left | 0.93 | 0.40 | 0.04 | 25.25 | -40.86 | -33.89 |
| RBANS List Learning | Left | 0.84 | 0.34 | 0.07 | -17.49 | 23.20 | -59.31 |
| RBANS List Recall | Left | 0.95 | 0.67 | <0.0001***** | -14.98 | -19.25 | -65.78 |
| RBANS Figure Recall | Left | 0.89 | 0.12 | 0.52 | -8.65 | -70.16 | -21.19 |
| RBANS Figure Copy | Left | 0.95 | 0.21 | 0.28 | 40.08 | 40.00 | 19.92 |
| SAS | Left | 0.92 | 0.17 | 0.41 | 39.02 | -59.85 | 1.13 |
| CES-D | Left | 0.71 | 0.15 | 0.45 | 48.75 | -50.19 | -1.07 |
| Apathy Evaluation scale | Left | 0.88 | 0.18 | 0.46 | -48.05 | 0.88 | 51.07 |

Hemi = hemisphere. LDA_1_ Prop = proportion of the total variance explained by the principle axis of variation as determined by LDA. *r* = Pearson’s *r*. p = unadjusted p-value. LDA_1_% refers to the percentage of the principle LDA axis that corresponds to each traditional MNI axis (X, Y, and Z) with sign indicating directionality. Note that per MNI convention, a negative X component indicates the medial direction for the right hemisphere but the lateral direction for the left. ***** = p-value is significant after false discovery rate correction.

**Table S8. Neuropsychological Tests Administered and Statistically Significant Results for Entire Cohort and Subcohorts based on Anxiety History.**

| TEST | PRIMARY DOMAIN | SECONDARY DOMAIN | cohort | no Anx | +anx |
| --- | --- | --- | --- | --- | --- |
| RBANS Digit Span Forward | Attention |  |  |  |  |
| WAIS Digit Span Backward | Attention | Executive |  | L |  |
| WAIS Picture Completion | Attention | Visuospatial |  | L |  |
| Color-Word Interference Test  (Stroop analogue) | Executive |  |  |  | L |
| Trail Making Test-B | Executive |  |  |  |  |
| WASI-II Matrix Reasoning | Executive | Visuospatial |  |  |  |
| WASI-II Similarities | Executive | Language |  | R |  |
| Controlled Oral-Word  association test (F-A-S) | Language | Executive |  |  | R |
| Boston Naming Test | Language | Visuospatial |  |  |  |
| RBANS Semantic Fluency | Language |  | L |  | R, L |
| Wechsler Test of Adult Reading  (WTAR) | Language |  |  |  |  |
| RBANS List Learning | Memory |  | L | R | L |
| RBANS List Recall | Memory |  | R, L | R, L | L |
| RBANS Figure Recall | Memory | Visuospatial |  |  |  |
| RBANS Figure Copy | Visuospatial |  |  |  |  |
| Zung Self-Rating Anxiety scale  (SAS) | Psychiatric |  |  |  |  |
| center for epidemiologic  studies depression scale (CES-D) | Psychiatric |  |  | R |  |
| Apathy Evaluation scale | Psychiatric |  |  |  |  |

Secondary cognitive domain only listed if applicable. ‘L’ and ‘R’ indicate statistically significant Pearson correlation between active contact location in the left (L) or right (R) hemisphere and change in postoperative test result.

**Table S9. LDA and Correlation Results for the History of Anxiety Subcohort.**

| TEST | hemi | LDA_1_ prop | *r* | p | LDA_1_ %X | LDA_1_ %Y | LDA_1_ %Z |
| --- | --- | --- | --- | --- | --- | --- | --- |
| RBANS Digit Span Forward | Right | 0.82 | 0.25 | 0.38 | -19.12 | -24.72 | -56.15 |
| WAIS Digit Span Backward | Right | 0.76 | 0.48 | 0.08 | -18.10 | 48.18 | 33.73 |
| WAIS Picture Completion | Right | 0.65 | 0.36 | 0.20 | 25.32 | 1.89 | 72.79 |
| Stroop analogue | Right | 0.87 | 0.15 | 0.58 | 26.83 | -0.32 | 72.85 |
| Trail Making Test-B | Right | 0.55 | 0.02 | 0.95 | 25.27 | 13.94 | 60.80 |
| WASI-II Matrix Reasoning | Right | 0.92 | 0.22 | 0.46 | -15.27 | -35.36 | -49.37 |
| WASI-II Similarities | Right | 0.80 | 0.37 | 0.22 | 4.82 | -9.94 | 85.24 |
| F-A-S | Right | 0.95 | 0.62 | 0.01***** | 1.12 | -13.46 | 85.42 |
| Boston Naming Test | Right | 0.86 | 0.42 | 0.12 | -33.39 | -37.69 | -28.92 |
| RBANS Semantic Fluency | Right | 0.99 | 0.83 | <0.001***** | -15.60 | -84.18 | -0.22 |
| WTAR | Right | N/A | N/A | N/A | N/A | N/A | N/A |
| RBANS List Learning | Right | 0.80 | 0.30 | 0.27 | -35.72 | -19.56 | -44.72 |
| RBANS List Recall | Right | 0.81 | 0.55 | 0.03 | 35.98 | -4.24 | 59.78 |
| RBANS Figure Recall | Right | 0.84 | 0.03 | 0.92 | -24.06 | -39.81 | -36.13 |
| RBANS Figure Copy | Right | 0.58 | 0.18 | 0.53 | -26.10 | -16.13 | -57.77 |
| SAS | Right | 0.61 | 0.59 | 0.02 | 19.75 | 16.69 | 63.56 |
| CES-D | Right | 0.90 | 0.19 | 0.51 | 29.00 | 32.54 | 38.46 |
| Apathy Evaluation scale | Right | 0.97 | 0.37 | 0.21 | -0.97 | -24.92 | -74.11 |
| RBANS Digit Span Forward | Left | 0.66 | 0.57 | 0.03 | -3.48 | -12.41 | -84.11 |
| WAIS Digit Span Backward | Left | 1.00 | 0.38 | 0.17 | -21.04 | 56.67 | 22.28 |
| WAIS Picture Completion | Left | 0.78 | 0.26 | 0.37 | 13.84 | -65.84 | -20.32 |
| Stroop analogue | Left | 0.76 | 0.70 | <0.01***** | -38.33 | -8.31 | -53.36 |
| Trail Making Test-B | Left | 0.90 | 0.43 | 0.11 | -50.72 | 33.86 | 15.42 |
| WASI-II Matrix Reasoning | Left | 0.80 | 0.03 | 0.92 | 69.57 | 15.22 | -15.22 |
| WASI-II Similarities | Left | 0.85 | 0.14 | 0.65 | -0.72 | -55.43 | -43.85 |
| F-A-S | Left | 0.79 | 0.00 | 1.00 | -25.72 | 48.22 | 26.06 |
| Boston Naming Test | Left | 0.64 | 0.41 | 0.13 | 14.02 | -32.31 | -53.67 |
| RBANS Semantic Fluency | Left | 1.00 | 0.74 | <0.01***** | 5.10 | -54.10 | -40.80 |
| WTAR | Left | N/A | N/A | N/A | N/A | N/A | N/A |
| RBANS List Learning | Left | 0.86 | 0.75 | <0.01***** | -59.26 | 27.77 | -12.98 |
| RBANS List Recall | Left | 0.90 | 0.66 | 0.01***** | 4.40 | 51.08 | 44.52 |
| RBANS Figure Recall | Left | 0.85 | 0.39 | 0.15 | 11.60 | -44.11 | -44.30 |
| RBANS Figure Copy | Left | 0.95 | 0.13 | 0.64 | 31.59 | 23.25 | -45.17 |
| SAS | Left | 0.98 | 0.34 | 0.21 | 31.38 | -59.79 | -8.83 |
| CES-D | Left | 0.90 | 0.18 | 0.54 | 74.92 | -15.96 | -9.12 |
| Apathy Evaluation scale | Left | 0.73 | 0.06 | 0.85 | -43.48 | 32.58 | 23.94 |

Hemi = hemisphere. LDA_1_ Prop = proportion of the total variance explained by the principle axis of variation as determined by LDA. *r* = Pearson’s *r*. p = unadjusted p-value. LDA_1_% refers to the percentage of the principle LDA axis that corresponds to each traditional MNI axis (X, Y, and Z) with sign indicating directionality. Note that per MNI convention, a negative X component indicates the medial direction for the right hemisphere but the lateral direction for the left. N/A = LDA unable to be run due to insufficient data range to establish three distinct performance classes as described in the methods section. ***** = p-value is significant after false discovery rate correction.

**Table S10. LDA and Correlation Results for the No History of Anxiety Subcohort.**

| TEST | hemi | LDA_1_ prop | *r* | p | LDA_1_ %X | LDA_1_ %Y | LDA_1_ %Z |
| --- | --- | --- | --- | --- | --- | --- | --- |
| RBANS Digit Span Forward | Right | 0.73 | 0.30 | 0.12 | -21.17 | -57.84 | -21.00 |
| WAIS Digit Span Backward | Right | 0.66 | 0.10 | 0.64 | 1.79 | -72.42 | 25.79 |
| WAIS Picture Completion | Right | 0.97 | 0.43 | 0.05 | 31.08 | -41.76 | -27.16 |
| Stroop analogue | Right | 0.98 | 0.31 | 0.17 | 19.40 | 24.62 | -55.98 |
| Trail Making Test-B | Right | N/A | N/A | N/A | N/A | N/A | N/A |
| WASI-II Matrix Reasoning | Right | 0.87 | 0.22 | 0.30 | -59.35 | -2.13 | -38.51 |
| WASI-II Similarities | Right | 0.97 | 0.54 | 0.01***** | 18.69 | -11.09 | -70.22 |
| F-A-S | Right | 0.90 | 0.25 | 0.22 | 5.53 | -51.23 | 43.25 |
| Boston Naming Test | Right | 0.87 | 0.03 | 0.87 | -18.89 | -31.13 | -49.99 |
| RBANS Semantic Fluency | Right | 0.85 | 0.16 | 0.43 | 60.47 | 26.15 | 13.38 |
| WTAR | Right | 0.86 | 0.28 | 0.20 | 39.30 | 52.67 | -8.03 |
| RBANS List Learning | Right | 0.91 | 0.47 | 0.02***** | 47.40 | 14.42 | -38.18 |
| RBANS List Recall | Right | 1.00 | 0.70 | <0.0001***** | 7.71 | -15.26 | -77.03 |
| RBANS Figure Recall | Right | 0.75 | 0.34 | 0.09 | -11.04 | -42.43 | -46.52 |
| RBANS Figure Copy | Right | 0.73 | 0.26 | 0.22 | -9.24 | -26.38 | -64.39 |
| SAS | Right | 1.00 | 0.45 | 0.03 | -26.95 | -36.36 | -36.69 |
| CES-D | Right | 0.61 | 0.49 | 0.02***** | -18.45 | -42.86 | -38.69 |
| Apathy Evaluation scale | Right | 0.74 | 0.05 | 0.85 | -19.66 | -34.44 | -45.90 |
| RBANS Digit Span Forward | Left | 0.81 | 0.32 | 0.10 | 2.27 | -44.15 | 53.58 |
| WAIS Digit Span Backward | Left | 0.70 | 0.67 | <0.001***** | -16.77 | 28.75 | 54.49 |
| WAIS Picture Completion | Left | 0.75 | 0.55 | 0.01***** | 39.54 | -24.38 | 36.07 |
| Stroop analogue | Left | 0.89 | 0.39 | 0.08 | 50.57 | -32.96 | -16.47 |
| Trail Making Test-B | Left | N/A | N/A | N/A | N/A | N/A | N/A |
| WASI-II Matrix Reasoning | Left | 0.98 | 0.30 | 0.16 | -15.73 | 11.87 | 72.40 |
| WASI-II Similarities | Left | 0.62 | 0.11 | 0.61 | 18.79 | -48.10 | -33.10 |
| F-A-S | Left | 0.90 | 0.41 | 0.04 | 33.79 | -49.43 | 16.78 |
| Boston Naming Test | Left | 0.98 | 0.32 | 0.11 | 39.66 | 30.42 | -29.92 |
| RBANS Semantic Fluency | Left | 0.93 | 0.32 | 0.10 | 14.97 | -17.96 | -67.07 |
| WTAR | Left | 0.90 | 0.38 | 0.07 | 4.44 | -47.72 | -47.84 |
| RBANS List Learning | Left | 0.91 | 0.27 | 0.19 | 40.09 | -16.78 | 43.13 |
| RBANS List Recall | Left | 0.97 | 0.51 | 0.01***** | -12.71 | -25.79 | -61.51 |
| RBANS Figure Recall | Left | 0.90 | 0.27 | 0.19 | -17.09 | -40.06 | 42.85 |
| RBANS Figure Copy | Left | 0.93 | 0.08 | 0.70 | 23.05 | 25.15 | 51.80 |
| SAS | Left | 0.79 | 0.39 | 0.06 | 2.85 | 48.34 | -48.81 |
| CES-D | Left | 0.75 | 0.18 | 0.41 | 48.98 | 20.26 | -30.75 |
| Apathy Evaluation scale | Left | 0.90 | 0.29 | 0.30 | 3.94 | 44.31 | -51.75 |

Hemi = hemisphere. LDA_1_ Prop = proportion of the total variance explained by the principle axis of variation as determined by LDA. *r* = Pearson’s *r*. p = unadjusted p-value. LDA_1_% refers to the percentage of the principle LDA axis that corresponds to each traditional MNI axis (X, Y, and Z) with sign indicating directionality. Note that per MNI convention, a negative X component indicates the medial direction for the right hemisphere but the lateral direction for the left. N/A = LDA unable to be run due to insufficient data range to establish three distinct performance classes as described in the methods section. ***** = p-value is significant after false discovery rate correction.

**Table S11. Subthalamic Nucleus Volumes of Tissue Activated**

| vta | ALL | DEPRESSION | ANXIETY | VH |
| --- | --- | --- | --- | --- |
| right Total | 144.2 (117.5) | 115.4 (96.0) | 132.5 (77.6) | 182.4 (125.2) |
| right proportion within STN | 0.76 (0.14) | 0.75 (0.14) | 0.81 (0.11)***** | 0.67 (0.19) |
| Right stn total | 108.6 (92.4) | 82.0 (58.1) | 109.1 (67.9) | 118.3 (81.9) |
| motor | 46.2 (41.8) | 33.0 (33.6) | 54.0 (37.0) | 48.1 (42.1) |
| associative | 50.5 (41.0) | 45.6 (36.7) | 52.3 (36.8) | 49.6 (39.9) |
| limbic | 27.5 (35.6) | 21.1 (29.4) | 30.7 (33.3) | 23.6 (27.8) |
| nonmotor | 78.1 (71.7) | 66.7 (64.8) | 83.0 (63.7) | 73.3 (62.5) |
| ratio of motor/nonmotor | 0.8 (0.8) | 0.7 (0.8) | 0.8 (0.7) | 1.1 (1.2) |
|  |  |  |  |  |
| left Total | 153.5 (113.7) | 136.7 (128.9) | 155.1 (102.6) | 207.1 (130.5) |
| left proportion within STN | 0.73 (0.19) | 0.74 (0.16) | 0.72 (0.21) | 0.68 (0.27) |
| left stn total | 111.9 (92.7) | 111.4 (121.8) | 114.4 (95.8) | 139.9 (118.6) |
| motor | 67.8 (62.7) | 59.5 (75.6) | 67.8 (69.9) | 88.7 (92.5) |
| associative | 34.7 (33.8) | 36.0 (50.5) | 30.3 (32.2) | 38.3 (33.7) |
| limbic | 44.3 (39.1) | 45.0 (52.1) | 43.0 (41.0) | 46.3 (52.7) |
| nonmotor | 79.0 (70.0) | 81.0 (99.1) | 73.4 (69.1) | 84.6 (85.2) |
| ratio of motor/nonmotor | 1.1 (1.0) | 1.1 (1.0) | 1.0 (0.7) | 1.2 (0.7) |

All values represent mean (SD) and are presented in mm^3^ (except for ‘proportion within STN’). Statistical comparisons are made between yes/no condition for depression, anxiety, and VH. Note that data for ‘no’ conditions are not shown for clarity. Instead, mean numbers for the entire cohort are also shown without any further statistical analysis. Nonmotor STN represents the sum of associative and limbic STN. VH = visual hallucinations. STN = subthalamic nucleus. VTA = volume of tissue activated. *p<0.05.

**Table S12. Correlation Between Ratio of Nonmotor to Motor Volume of Tissue Activated and Postoperative Neuropsychological Testing Changes.**

| TEST | hemi | Cohort | no dep | +DEP | no Anx | +anx |
| --- | --- | --- | --- | --- | --- | --- |
| RBANS Digit Span Forward | Right | -0.03 | 0.01 | -0.06 | 0.08 | -0.18 |
| WAIS Digit Span Backward | Right | -0.17 | -0.15 | -0.20 | -0.18 | -0.35 |
| WAIS Picture Completion | Right | 0.17 | 0.06 | 0.39 | 0.23 | 0.27 |
| Stroop analogue | Right | 0.19 | 0.03 | 0.58 | -0.01 | 0.48 |
| Trail Making Test-B | Right | 0.07 | 0.11 | 0.01 | 0.01 | 0.27 |
| WASI-II Matrix Reasoning | Right | 0.00 | -0.18 | 0.49 | -0.14 | 0.15 |
| WASI-II Similarities | Right | 0.07 | -0.01 | 0.15 | -0.05 | 0.39 |
| F-A-S | Right | 0.18 | 0.10 | 0.51 | 0.08 | 0.26 |
| Boston Naming Test | Right | -0.09 | 0.07 | -0.43 | -0.09 | 0.00 |
| RBANS Semantic Fluency | Right | 0.30 | 0.23 | 0.69**^#^** | 0.02 | 0.65**^##^** |
| WTAR | Right | 0.04 | 0.09 | 0.10 | -0.20 | 0.28 |
| RBANS List Learning | Right | -0.21 | -0.19 | -0.10 | -0.11 | -0.29 |
| RBANS List Recall | Right | 0.18 | 0.06 | 0.60 | 0.14 | 0.41 |
| RBANS Figure Recall | Right | 0.01 | 0.03 | 0.05 | 0.01 | 0.11 |
| RBANS Figure Copy | Right | 0.05 | 0.16 | -0.20 | -0.09 | 0.12 |
| SAS | Right | -0.30 | -0.11 | -0.75**^##^** | -0.38 | -0.31 |
| CES-D | Right | -0.23 | -0.08 | -0.47 | -0.26 | -0.18 |
| Apathy Evaluation scale | Right | -0.07 | 0.10 | -0.30 | -0.42 | 0.37 |
| RBANS Digit Span Forward | Left | 0.11 | 0.06 | 0.24 | 0.25 | -0.17 |
| WAIS Digit Span Backward | Left | -0.05 | -0.05 | -0.07 | 0.21 | -0.47 |
| WAIS Picture Completion | Left | 0.20 | 0.02 | 0.56 | 0.19 | 0.14 |
| Stroop analogue | Left | 0.10 | -0.06 | 0.40 | 0.29 | 0.06 |
| Trail Making Test-B | Left | -0.01 | -0.07 | 0.03 | 0.15 | -0.27 |
| WASI-II Matrix Reasoning | Left | 0.01 | -0.13 | 0.27 | 0.05 | 0.06 |
| WASI-II Similarities | Left | 0.03 | -0.16 | 0.27 | 0.05 | 0.12 |
| F-A-S | Left | 0.27 | 0.12 | 0.61**^#^** | 0.31 | 0.21 |
| Boston Naming Test | Left | -0.15 | -0.06 | -0.41 | -0.21 | -0.12 |
| RBANS Semantic Fluency | Left | 0.24 | 0.30 | 0.32 | -0.06 | 0.72***^##^** |
| WTAR | Left | 0.28 | 0.32 | 0.36 | 0.25 | 0.27 |
| RBANS List Learning | Left | -0.27 | -0.28 | -0.17 | -0.20 | -0.46 |
| RBANS List Recall | Left | 0.03 | -0.10 | 0.40 | -0.15 | 0.30 |
| RBANS Figure Recall | Left | -0.09 | 0.04 | -0.28 | -0.23 | 0.01 |
| RBANS Figure Copy | Left | 0.19 | 0.33 | -0.15 | 0.14 | 0.22 |
| SAS | Left | -0.34**^#^** | -0.23 | -0.56 | -0.28 | -0.46 |
| CES-D | Left | -0.13 | -0.04 | -0.05 | -0.22 | -0.12 |
| Apathy Evaluation scale | Left | -0.03 | 0.30 | -0.50 | -0.44 | 0.46 |

Spearman correlation between ΔT score and ratio of motor to nonmotor STN VTA, where nonmotor VTA is defined as the sum of associative and limbic STN VTAs. Hemi = hemisphere. STN = subthalamic nucleus. VTA = volume of tissue activated. *significant after correction for multiple comparisons, **^#^**uncorrected p<0.05, **^##^**uncorrected p<0.01.
